# Screening for Life-Threatening Arrhythmia in Asymptomatic Patients After Paediatric Cardiac Surgery: A Single-Centre Retrospective Analysis of 790 Pre-hospital-discharge 24-h Holter Electocardiogram Recordings

**DOI:** 10.1007/s00246-024-03691-7

**Published:** 2024-10-22

**Authors:** Evangelia Blana, Matthias Gass, Florian Berger, Hitendu Dave, Christian Balmer

**Affiliations:** 1 Pediatric Cardiology, Department of Surgery, Pediatric Heart Center, University Children’s Hospital, Steinwiesstr. 75, 8032 Zurich, Switzerland; 2 Children’s Research Centre, University Children’s Hospital Zurich, Zurich, Switzerland; 3 Lake Constance Heart Center, Luisenstr. 9, 78464 Constance, Germany; 4 Cardiothoarcic Surgery, Department of Surgery, Pediatric Heart Center, University Children’s Hospital, Steinwiesstr. 75, 8032 Zurich, Switzerland

**Keywords:** Congenital heart disease, Cardiopulmonary bypass, Severe arrhythmia, Risk factors, 24 h-ECG

## Abstract

Severe arrhythmias may occur early after open heart surgery. Because younger patients do not usually show any specific symptoms, presently Holter monitoring is routinely performed for 24 h predischarge at our centre to prevent adverse outcomes. It is unknown whether this test is truly justified in this patient population. Retrospective single-centre analysis of all consecutive patients younger than 19 years old after open heart surgery 2013–2019 who underwent routine Holter monitoring before hospital discharge. Patients with permanent pacemakers and patients who died during this hospital stay were excluded. The cohort was divided into two groups depending on whether severe arrhythmia occurred or not. The study includes 790 Holter recordings from 666 patients with a median age of 0.5 years (IQR 0.23–3.08), performed at a median time of 8 days (IQR 6–15) postoperatively. Postoperative arrhythmia was detected in 554 of 790 24-h Holter recordings (70%); in 47 of 790 (6%), this arrhythmia was classified as severe. The most common severe arrhythmias were premature ventricular contractions (*n* = 26/47) and long pauses (*n* = 14/47). A longer aortic cross-clamp time (mean 94.5 (SD ± 53.0) versus 68.1 (SD ± 51.9) min, *p* = 0.001) was associated with the occurrence of severe postoperative arrhythmia. Severe arrhythmias are rare in predischarge assessments after open heart surgery in children. In current postoperative monitoring at our centre, the diagnostic yield of ECG Holter monitoring for 24 h is too low to justify routine screening in all paediatric patients after open heart surgery.

## Introduction

Cardiac arrhythmias are a common phenomenon after congenital heart surgery. The reported incidence of postoperative arrhythmias in paediatric patients is 6.5% [1] and ranges up to 79.1% [2]. In the first days after open heart surgery, severe arrhythmias such as ventricular tachycardia or asystole may occur [1, 3–5].

Intermittent arrhythmias, such as short ventricular tachycardia and short episodes of atrioventricular block, can be a precursor to life-threatening events. However, these may be missed during the hospital stay. Younger patients may not show any specific symptoms. Thus, at the University Children’s Hospital Zurich, we have followed a policy of routine 24-h Holter monitoring to detect arrhythmias before hospital discharge. All postoperative patients are connected to a bed-side ECG monitor, not only during their intensive care unit stay but also on the ward, and the preparation, correct application, removal, and detailed evaluation of the 24-h ECG require substantial time and trained personnel. Consequently, determining whether the routine 24-h ECG recording adds benefit in this patient population may release resources for more effective therapeutic activities.

The aims of this study were (a) to assess the prevalence of severe arrhythmias in a paediatric cohort that is clinically ready to leave the hospital after cardiac surgery, (b) to define risk factors for the occurrence of severe arrhythmias in this context, and (c) to evaluate the usefulness of routine 24-h Holter monitoring in children after heart operations.

## Material and Methods

This is a retrospective analysis of all consecutive patients undergoing cardiopulmonary bypass surgery who underwent predischarge 24-h Holter monitoring between January 2013 and December 2019 at the University Children’s Hospital of Zurich. Patients were excluded from the study population if they were 19 years or older at time of surgery, they died due to cardiac or noncardiac complications during hospital stay, they had a permanent pacemaker, or their parents or legal guardians declined to consent to the use of their data. The study protocol was approved by the local ethical committee (KEK Zürich 2020-00613).

Holter monitoring is routinely performed for 24 h in all patients who undergo cardiopulmonary bypass surgery at our centre. The timing of this test depends on the individual course of the disease but is performed directly prior to hospital discharge. The timing ensures that the patient has fully recovered from the operation and has been fully mobilized. The electrodes are applied by a qualified nurse who has been trained for this task. The skin is carefully degreased, and each of the four electrodes is placed in a standard specified anatomical position. A tight-fitting elastic undershirt is then placed over the electrodes and cables on the chest to prevent the electrodes being displaced by patient movement. The recording ends after 24 h. It is then evaluated, first automatically using the software AMEDTEC® (08280 Aue, Germany) and then manually by an experienced paediatric cardiologist. The patient may only leave the hospital after the evaluation has been completed and approved.

The following data were obtained from patient records and from the electronic cardiology database: sex, age and weight at time of surgery, cardiac diagnosis, type of surgery, length of ICU stay, risk adjustment for congenital heart surgery (RACHS) score [6], cardiopulmonary bypass time, aortic cross-clamp time, lowest core temperature, and interval between surgery and 24-h Holter monitoring. Congenital heart defects were categorized into univentricular and biventricular heart defects by anatomical characteristics. The population was divided into newborns, comprising infants in the first 28 days after birth, and non-newborns, comprising all older patients. The high RACHS category was defined as including those with a RACHS score greater or equal to 4.

Premature atrial or ventricular contractions were divided into three groups according to their frequency: Rare (less than 240 beats per day), occasional (240 to 1440) and frequent (more than 1440). Less than 24 beats of premature atrial or ventricular contractions in 24 h were not classified as arrhythmia. Arrhythmias that might progress and result in syncope or sudden death were defined as severe, including second-degree type II AV block (type Mobitz), third-degree AV block, frequent premature ventricular contractions, (> 1440/24 h), occasional premature ventricular contractions (240–1440/24 h), junctional ectopic tachycardia, and long pauses, defined as pauses that are longer than two standard deviations from the norm [7]. A sustained tachycardia was defined a tachycardia that persists for more than 30 s [10, 11].

All other arrhythmias were considered non-severe and consisted of alterations in the heart rate or rhythm, including rare premature atrial or ventricular contractions (24–240/24 h), occasional premature atrial contractions (240–1440/24 h), frequent premature atrial contractions (> 1440/24 h), atrial rhythm, junctional or ventricular rhythm, premature junctional contractions, bradycardia, and tachycardia according to published normal limits [7].

In patients with severe arrhythmias, all retrospective data collected during the hospital stay was re-evaluated. Consideration was given to whether the diagnosis of a severe arrhythmia could have been made in another way if the 24-h Holter monitoring had been omitted.

Results are presented both as absolute numbers and relative frequencies. The comparison between the group with severe arrhythmia and the group without severe arrhythmia was made using the Chi-square test and Student’s *t* Test or the Mann–Whitney U test for categorical or continuous variables respectively. A *p* value < 0.05 was considered to be statistically significant. Multivariate logistic regression was used to determine potential risk factors for postoperative severe arrhythmias. Statistical analysis was performed using R Studio.

## Results

The study included 790 operations and subsequent 24-h Holter recordings in 666 patients. The 24-h ECG was recorded at a median time of 8 days (IQR 6–15 days) postoperatively. The median age and median body weight at the time of operation were 0.5 years (IQR 0.2–3.1 years) and 6.7 kg (IQR 4.5–13.5 kg) respectively. Further patient demographic and procedure-related characteristics are shown in Table 1. The cohort included 224 recordings in patients with single ventricle physiology (28.4%) and 137 recordings in newborns (17.3%).

**Table 1:** Patient characteristics; comparison of patients with and without severe arrhythmia

	Total Sample	No severe arrhythmia	Severe arrhythmia	*p*-Value
24-h Holter recordings, [n]	790	743	47	
Age at time of surgery, [years], median (IQR)	0.5 (0.2–3.1)	0.5 (0.2–3.1)	0.6 (0.3–3.8)	0.313
Age category newborns, [n], (%)	137 (17.3)	134 (18.0)	3 (6.4)	0.065
Weight at time of surgery [kg], median (IQR)	6.7 (4.5–13.5)	6.7 (4.50–13.4)	6.1 (5.0–15.1)	0.541
Male Sex, [n], (%)	455 (57.6)	431 (58.0)	24 (51.1)	0.434
Single ventricle physiology, [n], (%)	224 (28.4)	217 (29.2)	7 (14.9)	0.052
High RACHS^a^ category, [n], %	153 (19.4)	147(19.8)	6(12.8)	0.322
Aortic cross-clamp time [min], mean (SD)	69.7 (52.3)	68.1 (51.9)	94.5 (53.0)	**0.001**
Cardiopulmonary bypass time [min], mean (SD)	134.9 (70.4)	134 (70.6)	149 (65.3)	0.155
Lowest core temperature [°C], median (IQR)	32 (30–34)	32 (30–34)	32 (30–33)	0.683
ICU stay [days], mean (SD)	5.5 (10.9)	5.5 (11.2)	4.3 (4.4)	0.441
Interval between surgery and 24-h ECG [days], median (IQR)	8 (6–15)	9 (6–17)	7 (5–14)	0.275

Bold values indicate satistically significance P<0.05

Total number of patients: 666; 50 patients underwent two 24-h ECG recordings, 9 patients underwent three 24-h ECG recordings, 2 patients underwent four 24-h ECG recordings and 1 patient underwent five 24-h ECG recordings

^a^High RACHS category: RACHS category equal 4, 5 or 6

### Patients with Severe Arrhythmias

An arrhythmia was identified in 554 of 790 (70%) of the 24-h Holter recordings. A severe arrhythmia occurred in a total of 47 patients (6.0%). A detailed list of the findings is shown in Table 2. The most frequent severe arrhythmias were occasional premature ventricular contractions (*n* = 20) and long pauses (*n* = 14). The cardiac anatomy and perioperative condition of patients with severe cardiac arrhythmia (*n* = 47) were analysed in more detail. Severe arrhythmia occurred most frequently in patients with atrioventricular septal defect (*n* = 11), ventricular septal defect (*n* = 8), aortic valve stenosis (*n* = 5, with four undergoing repair and one undergoing replacement of the aortic valve), transposition of the great arteries (*n* = 4), atrial septal defect (*n* = 3), tetralogy of Fallot (*n* = 3), aortic valve regurgitation (*n* = 3, with one undergoing Ross procedure, one aortic valve reconstruction, and one aortic valve replacement), and Ebstein’s anomaly (*n* = 2). No specific pattern could be identified between the type of congenital heart defect or type of surgery and the occurrence of severe arrhythmias.

**Table 2:** Prevalence and type of arrhythmia

	n	%
Severe arrhythmia		
Second degree AV block type II (Mobitz type)	5	0.6
Long pause^a^	14	1.8
Frequent^b^ premature ventricular contractions	6	0.8
Occasional^c^ premature ventricular contractions	20	2.5
Junctional ectopic tachycardia	2	0.3
Non – severe arrhythmia		
AV reentrant tachycardia	1	0.1
Atrial ectopic tachycardia	15	1.9
Frequent^b^ premature atrial contractions	42	5.3
Occasional^c^ premature atrial contractions	54	6.8
Rare^d^ premature atrial contractions	195	24.7
Rare^d^ premature ventricular contractions	86	10.9
Premature junctional contractions	3	0.4
Junctional Rhythm	42	5.3

Values are shown as absolute numbers and percentage of the total number of 790 24-h Holter records. Ventricular tachycardia and complete atrioventricular block did not occur in this cohort

^a^Pauses were classified longer than two standard deviations of the norm [7]. Ectopic beats were categorized according to their frequency into, ^b^frequent i.e. more than 1440 beats; ^c^occasional i.e. 240 to 1440 beats or ^d^rare i.e. less than 240 beats per 24 h ECG recording

A junctional ectopic tachycardia was seen in two patients. In one of them, this arrhythmia had already been diagnosed on bedside monitoring on the intensive care unit.

Long pause was often associated with an atrioventricular septal defect (*n* = 5) and a ventricular septal defect (*n* = 4). In the majority of these 14 patients (*n* = 10), the long pause was associated with increased vagal tone, as judged retrospectively from clinical context, patient diary, and pre- and postpause ECG. In two patients, the pause was a compensatory pause after a premature contraction. Two ECGs with longer sinus pauses of 2.6 s and 2.3s cannot be assigned to either of these groups. In 4 out of 14 patients with long pauses on 24-h Holter monitoring, this finding could have been anticipated during the first postoperative days from bedside ECG monitoring with bradycardia (*n* = 1), ventricular tachycardia (*n* = 1) and advanced atrioventricular block (*n* = 2) requiring transient pacing.

Advanced atrioventricular block occurred in various heart defects, including bicuspid aortic valve with severe regurgitation (*n* = 1) undergoing Ross procedure, tetralogy of Fallot (*n* = 1), atrial septal defect (*n* = 1), atrioventricular septal defect (*n* = 1) and Ebstein's anomaly (*n* = 1) undergoing tricuspid valve reconstruction. The patient with Ebstein's anomaly had already developed a complete atrioventricular block intraoperatively, needing temporal pacing. On the other side, the patient with tetralogy of Fallot aroused our suspicion due to the pre-existence of a bifascicular block involving a right bundle branch block and a left anterior fascicular block. However, the remaining 3 of 5 patients with atrioventricular block did not show signs of this arrhythmia in previous postoperative monitoring. No cases of late complete atrioventricular block were observed. Notably, patients with a permanent pacemaker and therefore all patients with early postoperative complete heart block were excluded from this cohort.

Of the 20 patients with occasional premature ventricular contractions in the early postoperative phase, one patient developed hemodynamically relevant junctional ectopic tachycardia that required pacing, and one developed sinus bradycardia requiring pacing, while another one showed arrhythmia on bedside ECG monitoring (premature atrial contractions). All other patients had an uneventful early postoperative course regarding arrhythmia. One out of six patients with frequent premature ventricular contractions on predischarge 24-h Holter recording might have been identified previously from pre-existing ECG abnormalities. No sustained ventricular tachycardia occurred in our cohort. All patients remained asymptomatic.

In summary, in 11 of the 47 (23.4%) patients with severe arrhythmias, this arrhythmia could have been anticipated from early postoperative signs and symptoms and hints in the bedside ECG monitor, or in pre-existing pathologies like pre-existing arrhythmias.

### Risk Factor Analysis

Univariate analysis showed that a longer aortic cross-clamp time was a risk factor for the occurrence of life-threatening arrhythmias (94.5 vs. 68.1 min, *p* < 0.05). There was a trend towards a longer cardiopulmonary bypass time (149 vs. 134, *p* = 0.155) in patients with severe arrhythmia. Several parameters could be excluded as risk factors for severe arrhythmia: newborn age category, lower intraoperative core temperature, the presence of single-ventricle physiology, and a longer stay in the intensive care unit.

High RACHS category was not found to be a risk factor for developing a severe arrhythmia (12.8% vs. 19.8%, *p* = 0.322). Multivariate logistic regression was performed, including these parameters: gender, weight, aortic cross-clamp time, lowest core temperature and ICU-stay. Aortic cross-clamp time was the only parameter that was associated with the occurrence of severe arrhythmias (*p* = 0.001).

## Discussion

This study provides a unique opportunity to detail the prevalence of pre-hospital-discharge arrhythmias in children after open-heart surgery. To the best of our knowledge, this is the largest study to systematically analyse Holter ECG recordings of paediatric patients after cardiac surgery but before leaving the hospital. The numbers and types of arrhythmias documented in this cohort are clinically relevant, because the same arrhythmias can be expected in the early outpatient setting. Reassuringly, severe arrhythmias were detected in only 6% of our cohort on screening 24-h ECG recordings, and the two most serious postoperative arrhythmias, ventricular tachycardia and late complete AV block, did not occur.

Most postoperative arrhythmias are expected to occur within the first 24 h after cardiac surgery [4, 8]. Early postoperative arrhythmias following congenital cardiac surgery have been described to occur in 7% to 79% of paediatric patients [1–4, 9–12]. The highest incidence of 79% of postoperative arrhythmias is reported in a study that applied uninterrupted Holter ECG recording during the first 3 days after surgery [4]. Early postoperative arrhythmias are known to be transient [3, 4] and demonstrate low morbidity and mortality [2]. In our cohort, we did not focus on early postoperative arrhythmias but on arrhythmias occurring in the last days of the hospital stay at a median time of 8 days postoperatively. In fact, from the results of our study, we assume the majority of cardiac arrhythmias recorded right before hospital discharge is not followed by a critical clinical event. The prevalence of severe arrhythmias seemed to be lower at the time of recording a 24-h Holter monitoring, as the myocardium has had sufficient time to recover by then. The electrolyte imbalance induced by the use of cardioplegia and cardiopulmonary bypass also generally recovers well before the pre-discharge Holter recording. This could explain the different spectrum and frequency of arrhythmias observed at this time point. We found arrhythmias of all kinds, severe and non-severe, in 70% of the cohort, which confirms the high sensitivity of the 24-h Holter monitoring in this setting. In a recent study of 302 paediatric patients following cardiac surgery, an arrhythmia was detected in only 49% to 59% of postoperative 24-h Holter recordings [4]. However, that cohort is not directly comparable with the cohort used in this study because of differences in the definition of arrhythmias, age of patients, timing of the 24-h ECG, and surgical and ICU management strategies. The largest impact is from the differing definitions of arrhythmias. The additional consideration of pauses as arrhythmias and the broader definition of extrasystoles in our study may explain most of the discrepancy.

Recent studies have identified several risk factors for postoperative arrhythmia in children following heart surgery: younger age at surgery, higher RACHS score, prolonged cardiopulmonary bypass time, longer aortic cross-clamp time, longer intensive care-unit stay, and deep hypothermic circulatory arrest [5, 9, 11, 13, 14]. These risk factors are well understood from clinical practice. They are an indirect measure of myocardial vulnerability, myocardial trauma from non-physiological conditions during cardiopulmonary bypass and surgical instruments during surgery, and myocardial stress in postoperative low cardiac output syndrome. In our study, only prolonged aortic cross-clamp time posed a significant risk factor for severe arrhythmias (*p* < 0.05). There was a trend towards more severe arrhythmia in patients with longer cardiopulmonary bypass time, but this was not statistically significant. The age at surgery, RACHS category, length of ICU stay, and lowest core temperature were not clearly associated with higher rates of severe arrhythmia. The power of this analysis is limited by the low incidence of severe arrhythmias and the heterogeneity of the patient cohort. Also, the list of potential risk factors may be incomplete. In our study, repair of atrioventricular septum defect was the most common surgical procedure related to severe arrhythmias. This finding has also been reported in other cohorts [5, 12]. This is not surprising, because the repair of a complete atrioventricular septal defect involves several proarrhythmic factors, such as young age at the time of surgery, dilated atria, surgical manipulation near the bundle of His, and complex surgery with long cardiopulmonary bypass and cross clamp times. In addition, repeated testing of the left atrioventricular valve by water injection into the left ventricle may lead to significant microbubble air emboli. These factors subject patients to the risk of high-grade atrioventricular block and junctional ectopic tachycardia.

This retrospective study gives some evidence about the usefulness of routine 24-h Holter recording after cardiac surgery in a paediatric population to rule out a relevant arrhythmia: The 790 24-h recordings, which were taken at a median of 8 days postoperatively, contain only 47 severe arrhythmias, and none of them has the potential to be life-threatening. Moreover, 11 of these 47 arrhythmias (23%) would have been recognized earlier during the hospital stay. We hypothesize that many of the remaining arrhythmias would have been detected from the bedside patient ECG monitoring, which was not available for this retrospective analysis. We conclude that Holter monitoring for 24 h prior to discharge did not add significantly to patient safety in this cohort. To further address the question of the usefulness of routinely recording Holter monitoring for 24 h, a prospective study design including a blinded analysis of simultaneous Holter and bedside ECG monitoring would be desirable. The results of such a study would allow to include an individualized statement about the impact of the Holter findings on patient management and outcome in a specific patient situation.

The effort of routinely recording Holter monitoring for 24 h before discharge is not justified in patients who show no evidence of arrhythmia by symptoms, signs, or bedside ECG monitoring. We recommend an individual prescription of this test based on clinical criteria. Recorded Holter monitoring is a valuable adjunct to bedside ECG monitoring in selected cases. The risk factors reported, such as young age, complex cardiac surgery, and long times for cardiopulmonary bypass, aortic cross clamp, and ICU stay [5, 9, 11, 13], may help to guide the decision.

## Limitations

This study describes a cohort of relatively stable postoperative patients who are clinically ready to leave hospital. Furthermore, the time between surgery and 24-h Holter recording is relatively long and variable. Therefore, no conclusion can be drawn from our data about the overall incidence of postoperative arrhythmia in children after cardiac surgery. Generally, the power of this analysis is limited by the retrospective design of the study. Our sample is defined by consecutive cardiac operations at our centre and covers a wide range of ages and weights with a preponderance of patients in the lower weight categories. One of the key limitations of this study was the challenge associated with categorizing the ECG findings into more severe and less severe arrhythmia. Unfortunately, there are no generally applicable guidelines for this, which is also reflected in the different categorisation of arrhythmias in the studies published to date in this area [4, 10, 12]. The process of categorisation in our study was undertaken to the best of our knowledge and belief based on the authors' clinical experience and still remains subjective to a certain extent.

Risk factors for the occurrence of relevant arrhythmias cannot be reliably indicated only by age at surgery, time of aortic clamping, duration of cardiopulmonary bypass, lowest core temperature, and RACHS category. Obtaining an objective measure of surgical trauma to the myocardium requires detailed, individualized information on each patient, which we were unable to obtain in retrospect. Moreover, complex cardiac defects such as RACHS category 5 or 6 represented only a small proportion of our cohort (n = 33, 4.2%). Therefore, risk factors for severe arrhythmias, which are also rare events in our sample, are statistically difficult to identify. Only a very limited statement can be made about the occurrence of severe cardiac arrhythmias in older children and adolescents with our data, as the age distribution is shifted in favor of the younger children (median age 0.5 years).

## Conclusion

Our results show that cardiac arrhythmias are relatively common after congenital cardiac surgery in children, occurring even in the late postoperative period. However, in this selected retrospective cohort of paediatric patients who had recovered from their operation and were ready to leave the hospital, *severe* arrhythmias were a rare phenomenon.

We identified a long aortic cross clamping time as being associated with an increased incidence of severe arrhythmias on the 24-h Holter ECG recording before hospital discharge.

In this cohort, routine 24-h Holter recording did not have any impact on patient management or outcome. Therefore, predischarge 24-h ECG monitoring cannot be recommended as a screening tool in every paediatric patient after open heart surgery.

## Data Availability

Data sets generated during the current study are available from the corresponding author on reasonable request.

## References

[1] Sahu MK, Das A, Siddharth B, Talwar S, Singh SP, Abraham A, Choudhury A (2018) Arrhythmias in children in early postoperative period after cardiac surgery. World J Pediatric Congen Heart Surg 9(1):38–46. 10.1177/2150135117737687 10.1177/2150135117737687 29310559

[2] Pfammatter JP, Bachmann DCG, Wagner BP, Pavlovic M, Berdat P, Carrel T, Pfenninger J (2001) Early postoperative arrhythmias after open-heart procedures in children with congenital heart disease. Pediatr Crit Care Med 2(3):217–222. 10.1097/00130478-200107000-00005 12793944 10.1097/00130478-200107000-00005

[3] Alp H, Narin C, Baysal T, Sarıgül A (2014) Prevalence of and risk factors for early postoperative arrhythmia in children after cardiac surgery. Pediatr Int 56:19–23. 10.1111/ped.12209 24004418 10.1111/ped.12209

[4] Wortmann LG, Kreitz S, Grabitz RG, Vazquez-Jimenez JF, Messmer BJ, von Bernuth G, Seghaye MC (2010) Prevalence of and risk factors for perioperative arrhythmias in neonates and children after cardiopulmonary bypass: continuous holter monitoring before and for three days after surgery. J Cardiothor Surg 5:85 10.1186/1749-8090-5-85 PMC2974677 20955589

[5] Delaney JF, Moltedo JM, Dziura JD, Kopf JS, Snyder CS (2006) Early postoperative arrhythmias after pediatric cardiac surgery. J Thorac Cardiovasc Surg 131:1296–1301. 10.1016/j.jtcvs.2006.02.010 16733160 10.1016/j.jtcvs.2006.02.010

[6] Jenkins KJ, Gauvreau K, Newburger JW, Spray TL, Moller JH, Iezzoni LI (2002) Consensus-based method for risk adjustment for surgery for congenital heart disease. J Thorac Cardiovasc Surg 123:110–118. 10.1067/mtc.2002.119064 11782764 10.1067/mtc.2002.119064

[7] Salameh A, Gebauer RA, Grollmuss O, Vít P, Reich O, Janousek J (2008) Normal limits for heart rate as established using 24-hour ambulatory electrocardiography in children and adolescents. Cardiol Young 18:467–472. 10.1017/s1047951108002539 18634710 10.1017/S1047951108002539

[8] Chelo D, Ateba NS, Tantchou Tchoumi JC, Ngo Nonga B, Mve Mvondo C, Kingue S, Abena Obama MT (2015) Early postoperative arrhythmias after cardiac surgery in children at the Shisong Cardiac Center, Cameroon. Health Sci. 10.5281/hsd.v16i2.523

[9] Valsangiacomo E, Schmid ER, Schupbach RW, Schmidlin D, Molinari L, Waldvogel K, Bauersfeld U (2002) Early postoperative arrhythmias after cardiac operation in children. Ann Thorac Surg 74:792–796. 10.5606/2Ftgkdc.dergisi.2021.20366 12238841 10.1016/s0003-4975(02)03786-4

[10] Talwar S, Patel K, Juneja R, Choudhary SK, Airan B (2015) Early postoperative arrhythmias after paediatric cardiac surgery. Asian Cardiovasc Thorac Ann 23(7):795–801. 10.1177/0218492315585457 25972292 10.1177/0218492315585457

[11] Yıldırim SV, Tokel K, Saygılı B, Varan B (2008) The incidence and risk factors of arrhythmias in the early period after cardiac surgery in pediatric patients. Turk J Pediatr 50:549–553 19227418

[12] Reckawek J, Kansy A, Miszczak-Knecht M, Manowska M, Bieganowska K, Brzezinska-Paszke M, Szymaniak E, Turska-Kmiec A, Maruszewski P, Burczynski P, Kawalec W (2007) Risk factors for cardiac arrhythmias in children with congenital heart disease after surgical intervention in the early postoperative period. J Thorac Cardiovasc Surg 133:900–904. 10.1016/j.jtcvs.2006.12.011 17382623 10.1016/j.jtcvs.2006.12.011

[13] Jain A, Alam S, Viralam SK, Sharique T, Kapoor S (2019) Incidence, risk factors, and outcome of cardiac arrhythmia postcardiac surgery in children. Heart Views 20:47–52. 10.4103/heartviews.heartviews_88_18 31462958 10.4103/HEARTVIEWS.HEARTVIEWS_88_18 PMC6686607

[14] Peretto G, Durante A, Limite LR, Cianflone D (2014) Postoperative arrhythmias after cardiac surgery: incidence, risk factors, and therapeutic management. Cardiol Res Pract 2014:615987. 10.1155/2014/615987 24511410 10.1155/2014/615987 PMC3912619

