# Correction: Screening for Life-Threatening Arrhythmia in Asymptomatic Patients After Paediatric Cardiac Surgery: A Single-Centre Retrospective Analysis of 790 Pre-hospital-discharge 24-h Holter Electocardiogram Recordings

**DOI:** 10.1007/s00246-024-03709-0

**Published:** 2024-11-21

**Authors:** Evangelia Blana, Matthias Gass, Florian Berger, Hitendu Dave, Christian Balmer

**Affiliations:** 1 Pediatric Cardiology, Department of Surgery, Pediatric Heart Center, University Children’s Hospital, Steinwiesstr. 75, 8032 Zurich, Switzerland; 2 Children’s Research Centre, University Children’s Hospital Zurich, Zurich, Switzerland; 3 Lake Constance Heart Center, Luisenstr. 9, 78464 Constance, Germany; 4 Cardiothoarcic Surgery, Department of Surgery, Pediatric Heart Center, University Children’s Hospital, Steinwiesstr. 75, 8032 Zurich, Switzerland

**Correction: Pediatric Cardiology** 10.1007/s00246-024-03691-7

In this article the author’s name ‘Evangelia Blana’ was incorrectly written as ‘Evangelina Blana’.

The original article has been corrected.

